# Dedifferentiation of Neurons Precedes Tumor Formation in *lola* Mutants

**DOI:** 10.1016/j.devcel.2014.01.030

**Published:** 2014-03-31

**Authors:** Tony D. Southall, Catherine M. Davidson, Claire Miller, Adrian Carr, Andrea H. Brand

**Affiliations:** 1 The Gurdon Institute and Department of Physiology, Development and Neuroscience, University of Cambridge, Tennis Court Road, Cambridge CB2 1QN, UK

## Abstract

The ability to reprogram differentiated cells into a pluripotent state has revealed that the differentiated state is plastic and reversible. It is evident, therefore, that mechanisms must be in place to maintain cells in a differentiated state. Transcription factors that specify neuronal characteristics have been well studied, but less is known about the mechanisms that prevent neurons from dedifferentiating to a multipotent, stem cell-like state. Here, we identify Lola as a transcription factor that is required to maintain neurons in a differentiated state. We show that Lola represses neural stem cell genes and cell-cycle genes in postmitotic neurons. In *lola* mutants, neurons dedifferentiate, turn on neural stem cell genes, and begin to divide, forming tumors. Thus, neurons rather than stem cells or intermediate progenitors are the tumor-initiating cells in *lola* mutants.

## Highlights

• The BTB-Zn finger factor, Lola, maintains neurons in a differentiated state
• Lola represses neural stem cell genes and cell-cycle genes in postmitotic neurons
• In *lola* mutants, neurons dedifferentiate
• Dedifferentiated neurons are tumor-initiating cells in *lola* mutants

## Graphical Abstract

**Figure:**
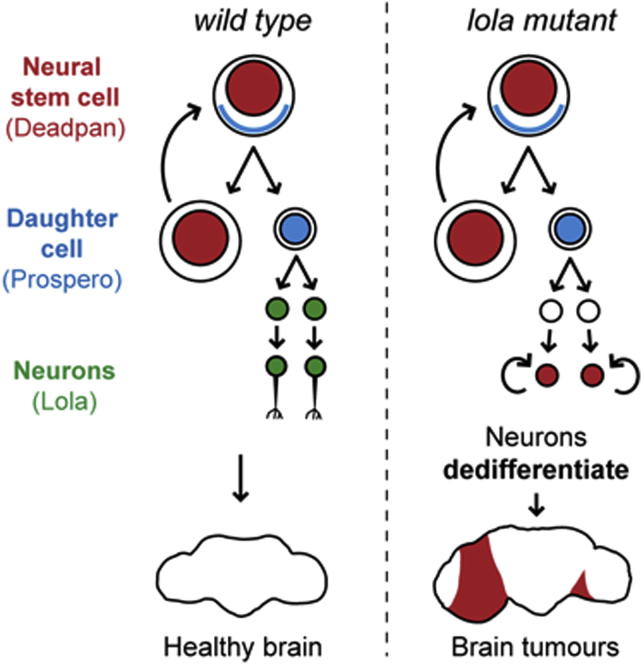

## Teaser

Southall et al. show that the BTB-Zn finger transcription factor, Lola, is required to maintain neurons in a differentiated state. Lola represses neural stem cell genes and cell-cycle genes in postmitotic neurons. In *lola* mutants, neurons dedifferentiate, turn on neural stem cell genes, and divide, forming tumors.

## Introduction

Waddington’s “epigenetic landscape” model suggests that the process of cellular differentiation is essentially irreversible (Waddington, 1957). However, it is now clear that differentiated cells can be reprogrammed into a pluripotent state (Gurdon, 1962; Takahashi and Yamanaka, 2006) or into an alternative differentiated state (Vierbuchen et al., 2010). Therefore, the differentiated state of cells is not set in stone; cells can dedifferentiate or transdifferentiate. However, the more differentiated a cell is, the more difficult it is to reprogram (for review, see Pasque et al., 2011), suggesting that there are active mechanisms in place to maintain cells in a differentiated state. Although there are now many studies defining the transcription factors that enable reprogramming, less is known about the mechanisms that act to prevent cells from dedifferentiating. Identification of these mechanisms will be key for fully understanding reprogramming and for developing safe methods for dedifferentiating cells in vivo for therapeutic purposes without inducing cancer (van Es et al., 2012; Schwitalla et al., 2013).

The *Drosophila* CNS is an attractive model for studying differentiation of neural stem cells and their progeny as well as investigating how misregulation of self-renewal and differentiation can lead to tumorigenesis (Wodarz and Gonzalez, 2006; Caussinus and Hirth, 2007; Doe, 2008; Egger et al., 2008; Neumüller and Knoblich, 2009). The majority of neural stem cells in the *Drosophila* brain and ventral nerve cord (type I neuroblasts) undergo multiple asymmetric divisions whereby they self-renew while producing daughter cells (GMCs or ganglion mother cells) that divide only once to give two postmitotic neurons or glial cells. At each division, cell-fate determinants are segregated from the neural stem cell to the GMC. These include Prospero (Doe et al., 1991; Vaessin et al., 1991; Matsuzaki et al., 1992), Brat (Bello et al., 2006; Betschinger et al., 2006; Lee et al., 2006b), and Numb (Rhyu et al., 1994), all of which act as tumor suppressors in the nervous system (Bello et al., 2006; Betschinger et al., 2006; Choksi et al., 2006; Lee et al., 2006a, 2006b; Wang et al., 2006; Bowman et al., 2008). Therefore, disrupting the neurogenic differentiation pathway can lead to tumorigenesis.

We showed previously that the atypical homeodomain transcription factor, Prospero, controls the choice between stem cell self-renewal and differentiation. Prospero represses genes required for self-renewal, such as neural stem cell genes and cell-cycle genes but also is required to activate genes for neuronal and glial differentiation (Choksi et al., 2006). In *prospero* mutants, GMCs fail to differentiate and revert to a stem cell-like fate. They continue to divide, express neural stem cell markers, and form brain tumors (Caussinus and Gonzalez, 2005; Bello et al., 2006; Betschinger et al., 2006; Choksi et al., 2006; Lee et al., 2006b).

Prospero’s ability both to repress and to activate transcription suggested that cofactors and/or chromatin remodeling factors might modulate Prospero’s activity. Prospero is known to function with a histone deacetylase (HDAC), Rpd3, to control dendritic targeting in postmitotic neurons (Tea et al., 2010). The vertebrate homolog of Prospero, Prox1, also interacts with an HDAC (HDAC3; Shan et al., 2008) as well as with nuclear hormone receptors (Liu et al., 2003; Steffensen et al., 2004; Lee et al., 2009; Charest-Marcotte et al., 2010). To date, no factors have been identified that act with Prospero in the switch from self-renewal to differentiation.

Here, we show that the BTB-Zn finger transcription factor, Lola (Seeger et al., 1993; Giniger et al., 1994), binds to a large number of Prospero’s targets, including genes involved both in stem cell self-renewal and differentiation. Furthermore, like Prospero, Lola is a tumor suppressor protein. Intriguingly, however, the tumor cell of origin in *lola* mutants differs from that in *prospero* mutants. Unlike *prospero* mutants where the first daughter of the neural stem cell, the GMC, reverts to a stem cell-like state, in *lola* mutants newly born neurons dedifferentiate, express stem cell markers, and proliferate, resulting in brain tumors. We conclude that, whereas Prospero acts to block self-renewal and initiate neuronal differentiation, Lola is required to maintain the differentiated state.

## Results

### Identification of a Prospero Cofactor

**Figure 1:**
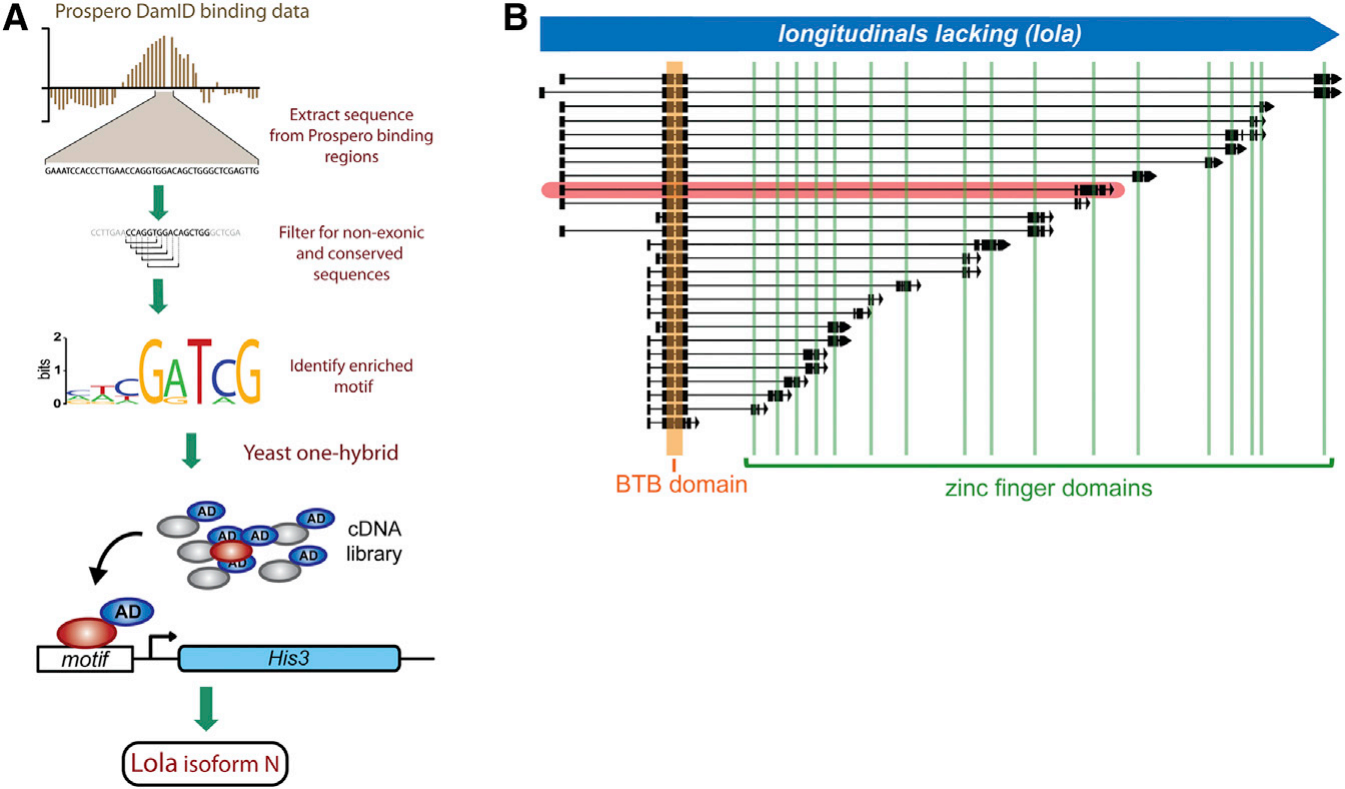
Experimental Design for Identifying a Prospero Cofactor (A) An enriched motif was identified at regions in the genome bound by Prospero. In the Prospero DamID binding data example, the vertical bar represents the log2 ration between the Dam-Prospero signal and the Dam-only signal. This motif identified was used in a yeast one-hybrid screen to identify Lola splice isoform N as a protein that can bind to this DNA sequence. (B) Structure of the *lola* locus. *lola* generates 25 different splice isoforms that all share a N-terminal BTB domain but possess one of 17 differing C-terminal zinc finger domains. The isoform identified in the yeast one-hybrid screen is highlighted (Lola-N).

Given that Prospero has the ability both to repress and activate gene expression (Choksi et al., 2006), we reasoned that Prospero is likely to act with proteins that are able to modulate its activity. To identify the binding sites for potential cofactors, we analyzed the DNA sequences near Prospero binding sites using the motif discovery tool MICRA (Southall and Brand, 2009). MICRA identifies enriched sequences at Prospero binding sites by extracting and filtering for conserved sequences and calculating the relative frequency of each 6–10-mer as compared to the background frequency throughout the genome. The most enriched 6-mer at Prospero binding sites, to which Prospero itself does not bind (Cook et al., 2003; Yousef and Matthews, 2005), is the conserved palindromic sequence, CGATCG (166% enriched). Furthermore, alignment of the most enriched 8-mer generates a position weight matrix (PWM) containing a core CGATCG sequence (Figure 1A). Members of the GATA zinc finger transcription factor family (Bryne et al., 2008) bind a similar sequence (GATDV, GATYDD).

To identify proteins that recognize this motif, we performed a yeast one-hybrid screen using six copies of the motif as bait and an embryonic cDNA library as prey. We isolated a specific isoform of the BTB-zinc finger transcription factor Lola, Lola-N. The *lola* locus generates 25 different splice isoforms encoding 20 proteins that share the same N-terminal BTB domain but different C termini, which can encode one of 17 different zinc fingers, or lack zinc fingers entirely (Goeke et al., 2003; Ohsako et al., 2003) (Figure 1B). Each of these zinc fingers can potentially bind a unique DNA sequence.

### Lola-N Is Expressed in Neurons

**Figure 2:**
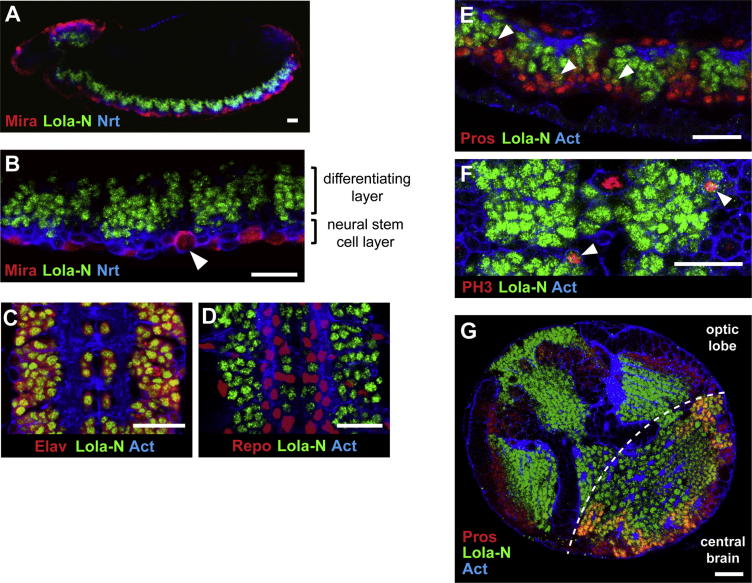
Lola-N Is Expressed in Neurons in the Developing Nervous System (A and B) Lola-N protein is absent from neuroblasts and present in the differentiating, dorsal region of the VNC (lateral view, stage 14 embryo). Arrowhead highlights a neuroblast. (C) Colocalization of Lola-N and the neuronal marker Elav (ventral view, stage 16/17 embryo). (D) Mutually exclusive expression pattern of Lola-N and the glial marker Repo (ventral view, stage 16/17 embryo). (E) Lola-N and Prospero briefly overlap in differentiating cells of the VNC (lateral view, stage 14 embryo). Arrowheads identify example cells that express both Lola-N and Prospero. (F) Colocalization of low levels of Lola-N with PH3 in the VNC (ventral view, stage 13 embryo). Arrowheads show dividing GMC cells. (G) Lola-N and Prospero expression in third instar larval brains. Scale bars represent 20 μM. See also Figure S1.

If Lola acts with Prospero, then we expected that the two proteins would colocalize at some point in development. To determine the expression pattern of Lola-N, we generated an antibody against its unique C terminus. Lola-N is expressed in embryos from stage 11 onward in the differentiating layer of the ventral nerve cord (VNC) but not in neuroblasts (Figures 2A and 2B). Lola-N is expressed in all neurons, colocalizing with Elav, but is undetectable in glial cells (Figures 2C and 2D). Lola-N is also expressed in neurons of the larval and adult brain (Figure 2G and Figures S1A–S1G available online).

During neural stem cell self-renewal, Prospero is segregated from the neuroblast to the GMC. After the GMC has divided, Prospero is present only transiently in the resultant neurons (Spana and Doe, 1995). Lola-N expression is induced as Prospero levels decrease, resulting in a brief period of colocalization (Figure 2E) immediately before, and just after, GMC division, as determined by phosphohistone H3 (PH3) labeling (arrowheads in Figure 2F).

Lola-N is expressed in a similar manner in the larval CNS (Figures 2G and S1A–S1C), except that it overlaps more extensively with Prospero in the central brain (Figure S1C) where Prospero is present in at least a subset of postmitotic neurons (Bello et al., 2006; Tea et al., 2010). Therefore, Lola-N is expressed at an appropriate time and place to act with Prospero or to modulate its activity.

### Lola-N Represses Neuroblast Genes and Cell-Cycle Regulators

**Figure 3:**
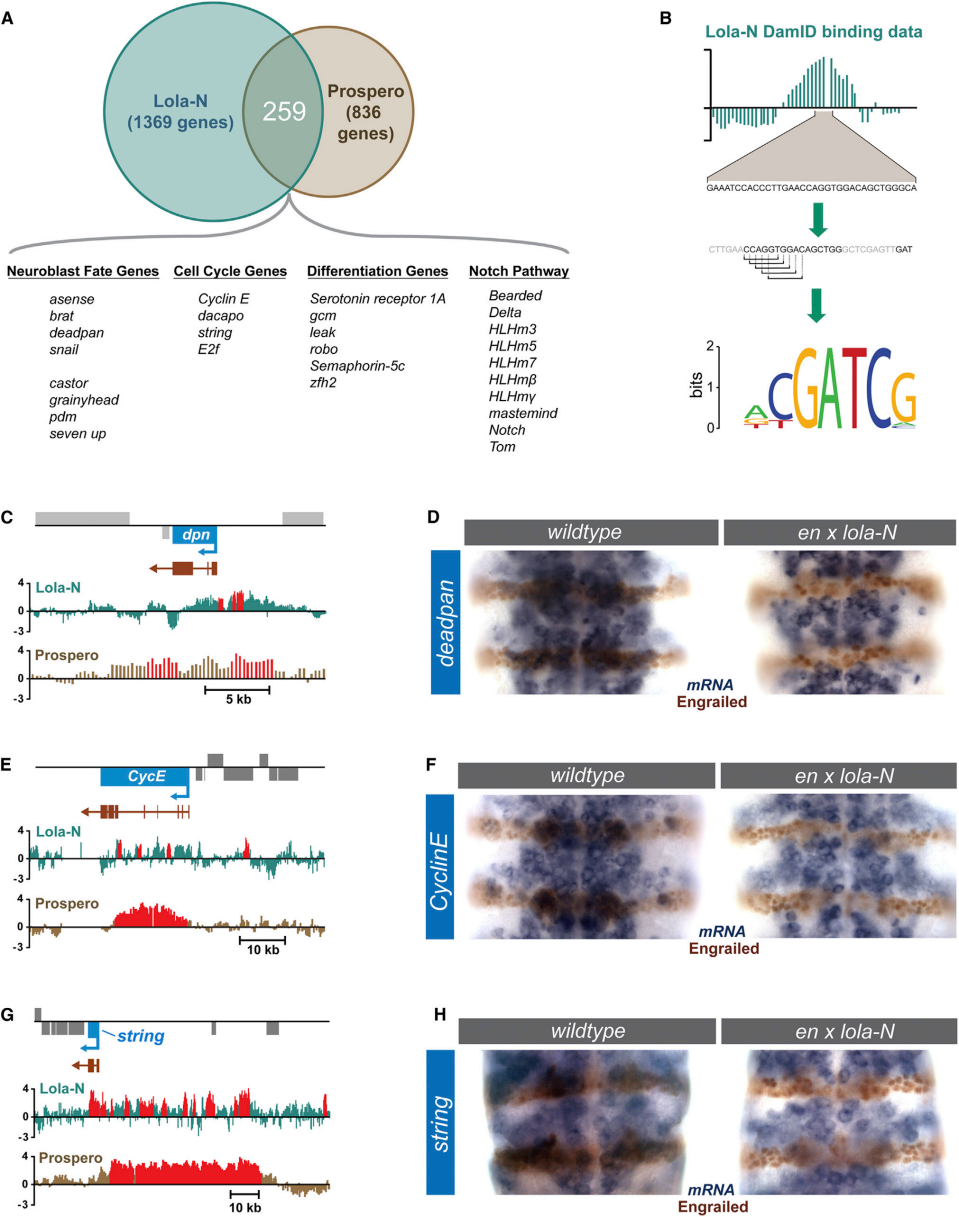
Transcriptional Targets of Lola-N (A) Comparison of Lola-N and Prospero target genes in the developing embryo. (B) Identification of the most enriched DNA motif at sites of Lola-N binding. In the Lola-N DamID binding data example, the vertical bar represents the log2 ratio between the Dam-Prospero signal and the Dam-only signal. (C, E, and G) Lola-N and Prospero binding at the *dpn*, *CycE*, and *string* loci. The vertical bars represent the log2 ration between the Dam-fusion signal and the Dam-only signal. Red bars indicate regions identified as being significantly bound. (D, F, and H) Lola-N is sufficient to repress the expression of *dpn*, *CycE*, and *string* mRNA in the developing embryo. See also Figure S2.

To assess whether Lola binds to the same genes as Prospero, we identified the embryonic binding sites of Lola-N throughout the genome using DamID. Lola-N binds to 1,369 genes (false discovery rate [FDR] < 0.1%) that show a highly significant overlap (p < 6 × 10^−71^) with the 836 genes (FDR < 0.1%) bound by Prospero. Two hundred fifty-nine genes are bound by both Prospero and Lola-N (31% of Prospero’s targets; Figure 3A; Experimental Procedures). This overlap is specific to Prospero and Lola-N because only five of the 259 genes (2%) are bound by an unrelated neural transcription factor (P. Wu and A.H.B., unpublished data).

Analysis of the Lola-N binding peaks reveals that the most enriched 6-mer (CGATCG, 253% enriched) is identical to the motif identified by MICRA analysis of the sequences associated with Prospero binding peaks, as described above. The core CGATCG sequence is again integral to a PWM generated from enriched 8-mer (Figure 3B) and provides independent support for the yeast one-hybrid result.

Lola-N binds both neural stem cell genes and differentiation genes (Figure 3A). Both Lola-N and Prospero bind key neuroblast genes and cell-cycle regulators (Figures 3C, 3E, and 3G), including *brain tumor* (*brat*), *deadpan* (*Hes* family related), *dacapo* (*p27cip/kip*), and *string* (*cdc25*). They also bind to many Notch family genes, suggesting that Prospero and Lola-N coordinately regulate this pathway. Interestingly a second isoform of Lola, Lola-T, has been shown to antagonize Notch during specification of cell fate in the developing *Drosophila* eye (Zheng and Carthew, 2008).

To determine how Lola-N regulates its target genes, we expressed Lola-N ectopically in stripes in the developing embryo. In cells expressing Lola-N, driven by *engrailed-GAL4*, transcription of the cell-cycle genes *CyclinE* and *string* (*cdc25*) is repressed (Figures 3F and 2H). Transcription of the genes encoding the neuroblast transcription factors, *deadpan* and *asense*, is also repressed by Lola-N (Figures 3D, S2A, and S2B). Therefore, like Prospero, Lola-N is able to directly bind and repress neural stem cell genes. This is intriguing as Lola-N is first expressed just prior to the GMC’s terminal division (Figure 2F), at which point Prospero would already have repressed neural stem cell genes. This suggests that Lola-N’s role might be in maintaining, rather than in initiating, repression of these genes.

### Loss of *lola* Causes Tumors

If Lola-N is required to maintain the differentiated state, then the loss of Lola might result in tumor formation, similar to what has been observed in *prospero* mutants. Prospero is required to block self-renewal and induce differentiation. As a result, the loss of *prospero* leads to tumor formation in the developing *Drosophila* nervous system. In *prospero*, mutant GMCs, which would normally divide terminally to generate postmitotic neurons, instead undergo self-renewing divisions (Choksi et al., 2006; Lee et al., 2006b).

**Figure 4:**
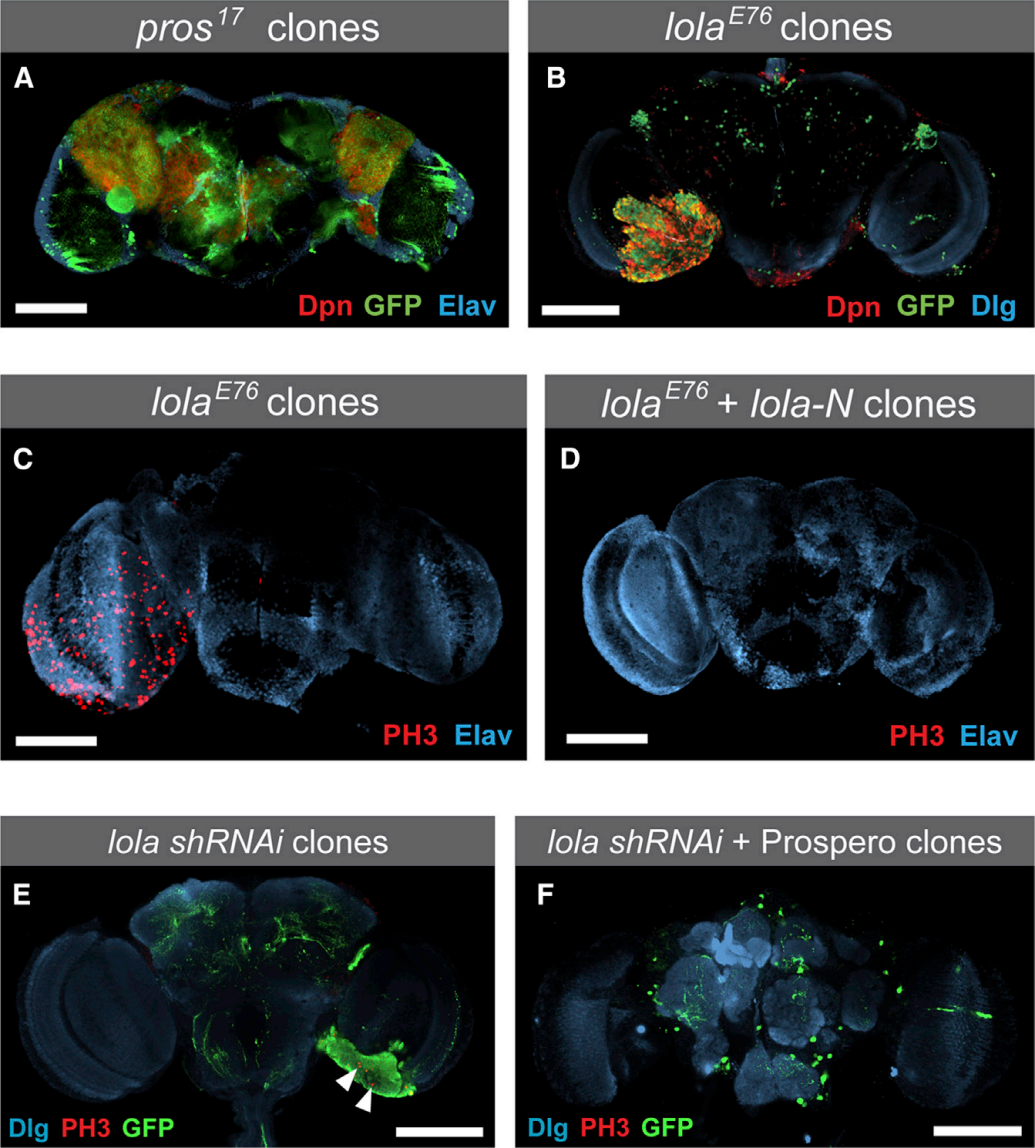
Loss of Lola Causes Brain Tumors (A) *prospero* mutant clones result in tumors in the adult brain, in both the central brain and optic lobe regions. (B and C) *lola*^*E76*^ mutant clones cause tumors in the optic lobe regions of the adult brain. The tumors express the neuroblast marker Deadpan (B) and contain actively dividing cells (PH3; C). (D) Expression of Lola-N in *lola*^*E76*^ mutant clones is sufficient to rescue the tumor phenotype. (E) actin FLP-out clones that express *lola shRNAi* (induced during the second instar larval stage) result in tumors in the adult brain (five out of nine brains). Arrowheads highlight dividing cells (PH3). (F) FLP-out clones that express both *lola shRNAi* and Prospero do not cause tumors (11 out of 11 brains). Clones are marked with GFP. Scale bars represent 100 μM. See also Figure S3.

We generated *lola*^*E76*^ mutant clones during larval stages using the MARCM system (Lee and Luo, 2001). *lola*^*E76*^ is a protein null mutation, removing all isoforms of *lola* (Goeke et al., 2003). *lola*^*E76*^ mutant clones proliferate extensively and give rise to tumors in the adult brain (Figures 4B and 4C). The mutant cells express the neuroblast transcription factor, Deadpan (Figure 4B), and divide actively as indicated by labeling with phosphohistone H3 (Figure 4C). We observed a similar phenotype with an independent *lola* mutant allele, *lola*^*5D2*^ (Giniger et al., 1994) (data not shown). *lola*^*E76*^ mutant clones form tumors in the optic lobe region of the adult brain (n > 30) but not in the central brain, in contrast to *prospero* mutant clones, which can generate tumors in both regions (Bello et al., 2006; Betschinger et al., 2006; Lee et al., 2006b) (Figure 4A). Interestingly, Prospero expression persists in the central brain but not in the optic lobe. It is conceivable that Prospero is able to maintain the repression of neural stem cell genes in the central brain in the absence of Lola. In support of this hypothesis, ectopic expression of Prospero is able to rescue the *lola* tumor phenotype (Figures 4E and 4F).

A previous study described the formation of tumors in the larval central brain when *lola* was knocked down using RNAi (Neumüller et al., 2011). However, RNAi is prone to off-target effects. We have never observed tumor formation in the central brain in two different *lola* null mutants (*lola*^*E76*^ and *lola*^*5D2*^), nor after expression of a small hairpin RNA (shRNA) (Ni et al., 2011) targeting *lola* (Figure S6A). The same study reported a neuroblast underproliferation phenotype after knockdown of *lola-N* in neuroblasts. As *lola-N* is not expressed in neuroblasts, this is likely also to be due to off-targets.

To test whether the loss of Lola-N is critical for tumor formation, we expressed *lola-N* in *lola*^*E76*^ mutant clones. Expression of *UAS-lola-N* was driven by *elav-GAL4* to most closely replicate wild-type *lola-N* expression. *lola*^*E76*^ clones expressing *lola-N* never form tumors (nine of nine brains; Figure 4D). In contrast, neither Lola-F nor Lola-H is able to inhibit tumor formation (C. Howard, T.D.S., and A.H.B., unpublished data). Interestingly, ectopic expression of Lola-N is also able to suppress the *prospero* mutant phenotype in embryos, preventing Deadpan expression and cell proliferation (Figure S3). Therefore, like Prospero, Lola-N acts as a tumor suppressor.

### Neurons Lacking *lola* Dedifferentiate

Lola-N is expressed in postmitotic neurons, implying that there is active repression of stem cell genes in postmitotic neurons and raising the possibility that *lola* mutant tumors arise through the dedifferentiation of neurons. To test this hypothesis, we investigated the timing of tumor formation and the cell type of origin in *lola* mutants.

**Figure 5:**
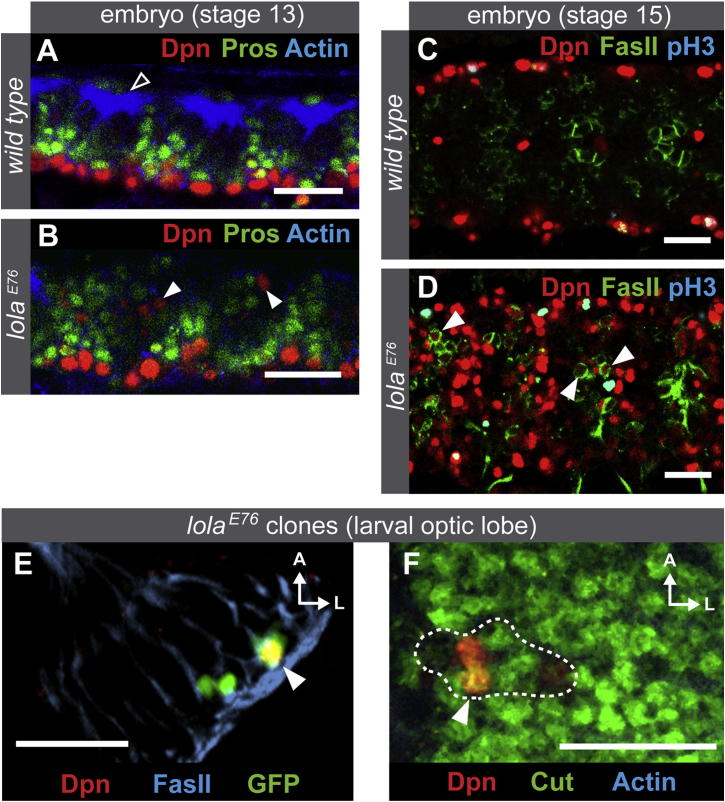
In *lola* Mutants, Neuroblast Genes Are Switched Off in GMCs but Reexpressed in Neurons (A) In wild-type embryos Deadpan is switched off in GMCs and remains off. (B) In lolaE76 embryos Deadpan is switched off normally in GMCs; however, ectopic Deadpan is observed in the dorsal more differentiated region of the VNC (see filled arrowheads). Less organized actin structures are observed in lolaE76 embryos, compared to wild-type (see empty arrowhead), due to disruption of axonal projections. (C) Expression of Deadpan and FasII in wild-type stage 15 embryos. Ventral view at the level of midline neuroblasts. (D) Deadpan and FasII expression in lola mutant embryos. Arrowheads show cells expressing both Deadpan and FasII. (E) Coexpression of Deadpan and FasII in lola mutant clones in the developing optic lobe. Arrowheads show cells expressing both Deadpan and FasII. (F) Coexpression of Deadpan and Cut in lola mutant clones in the developing optic lobe. Arrowheads show cells expressing both Deadpan and Cut. Scale bars represent 20 μM. See also Figure S4.

In *prospero* mutants, tumors arise from GMCs that revert to a stem cell-like fate, expressing neuroblast genes such as Deadpan (Choksi et al., 2006; Lee et al., 2006b). In wild-type embryos, Deadpan is expressed in neuroblasts, which lie ventrally (red cells in Figure 5A). When Prospero moves into the nucleus of GMCs, Deadpan expression is rapidly repressed (green cells in Figure 5A). In *lola* mutant embryos, Deadpan is switched off normally in GMCs (Figure 5B) but is then ectopically expressed in the dorsal, differentiated layer of the VNC, where neurons are positioned (arrowheads in Figure 5B). These cells are not GMCs or newly born neurons as they do not express Prospero (Figure 5B). To confirm that these cells are neurons, we costained with Fasciclin II, which is expressed in neurons but not neuroblasts (Kristiansen and Hortsch, 2010). In wild-type embryos, Deadpan (neuroblasts) and Fasciclin II (neurons) are never coexpressed (Figure 5C); however, *lola* mutant neurons express both Fasciclin II and Deadpan, both in the embryo (Figure 5D) and in the larval optic lobe (Figure 5E). We observed Deadpan coexpressed with two further neuronal markers, Cut and Elav (arrowheads in Figures 5F and S4). Therefore, Deadpan is properly repressed in GMCs but is then derepressed in neurons.

**Figure 6:**
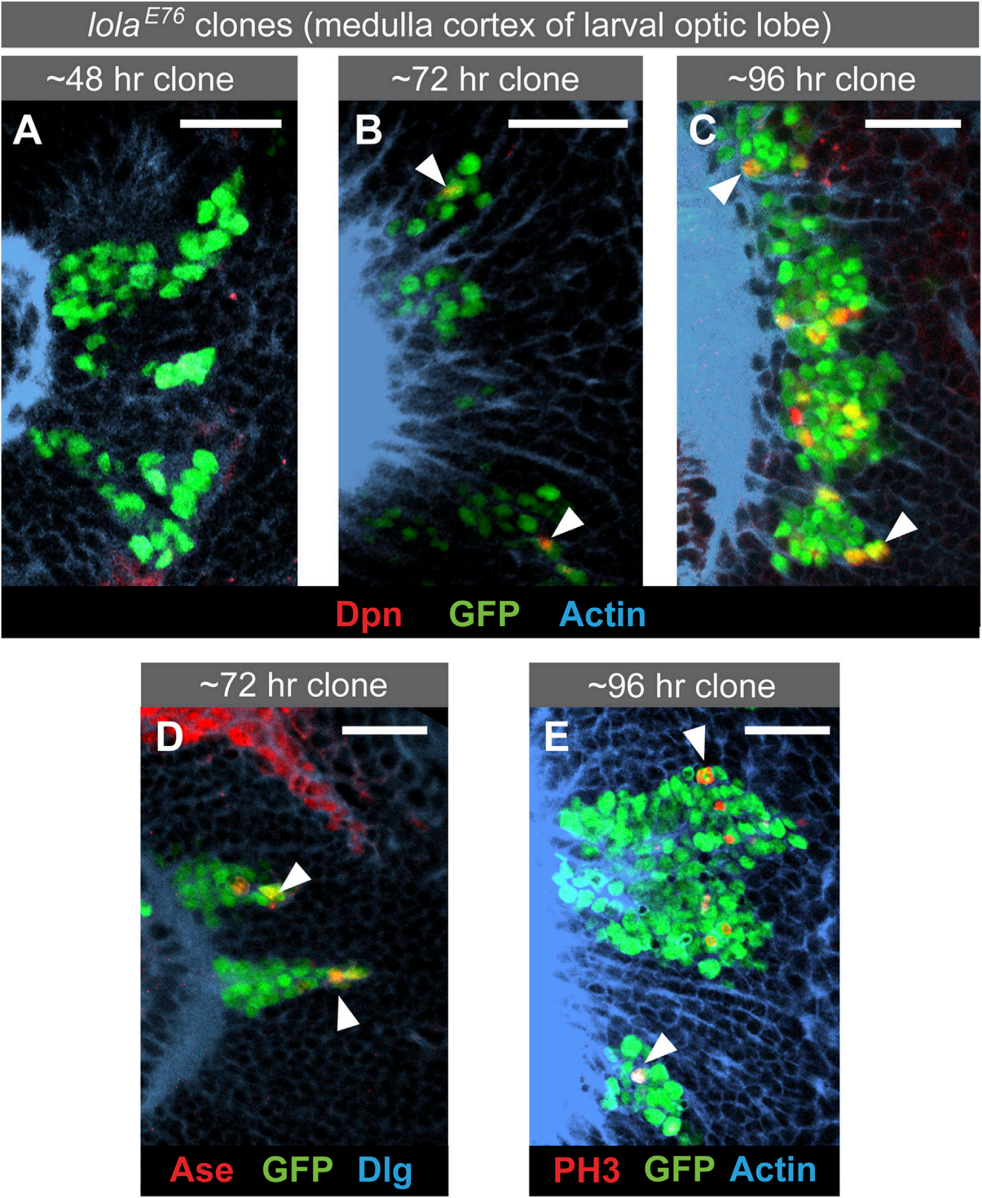
Time Course of Dedifferentiation in *lola* Tumors (A–C) Time course of dedifferentiation in *lola* tumors. In the outer medulla cortex of the developing optic lobe, ectopic Deadpan is not observed until ∼72 hr after clone induction (see arrowheads). (D) Ectopic expression of the neuroblast gene Asense (see arrowheads). (E) Ectopic division (PH3) is observed ∼96 hr after clone induction (see arrowheads). Scale bars represent 20 μM. See also Figure S5.

Next, we followed the progression over time of *lola*^*E76*^ mutant clones in the larval optic lobe (Figures 6A–6C and S5C). The timing and position of the Deadpan-expressing cells provided further confirmation that neurons, rather than GMCs, are the tumor-initiating cells. Initially, we observed only neurons in *lola* mutant clones in the medulla cortex (∼48 hr; only 1% of *lola* mutant medulla cortex cells show Deadpan expression). By ∼72 hr, Deadpan began to be expressed in regions of the medulla cortex where only postmitotic neurons normally reside (5% of *lola* mutant cells) (Figure 6B). By ∼96 hr, multiple Deadpan-positive cells were found in mutant clones (17%) in the differentiated outer medulla cortex (Figure 6C). We observed ectopic expression of two further neuroblast genes, Asense and Worniu, in the medulla cortex (Figures 6D and S5A). The expression of neuroblast genes coincides with cells entering the cell cycle and actively dividing (Figure 6E). We observe PH3-positive cells in deep layers of the medulla cortex, indicating that the *lola* mutant cells are actively proliferating in a region of the brain where there is normally little or no cell division (Figure 6E, compare *lola* mutant cells to surrounding wild-type neurons). Consistent with us never observing tumors in the adult central brain, ectopic Deadpan is not present in *lola* mutant clones in the larval central brain (Figure S5B).

**Figure 7:**
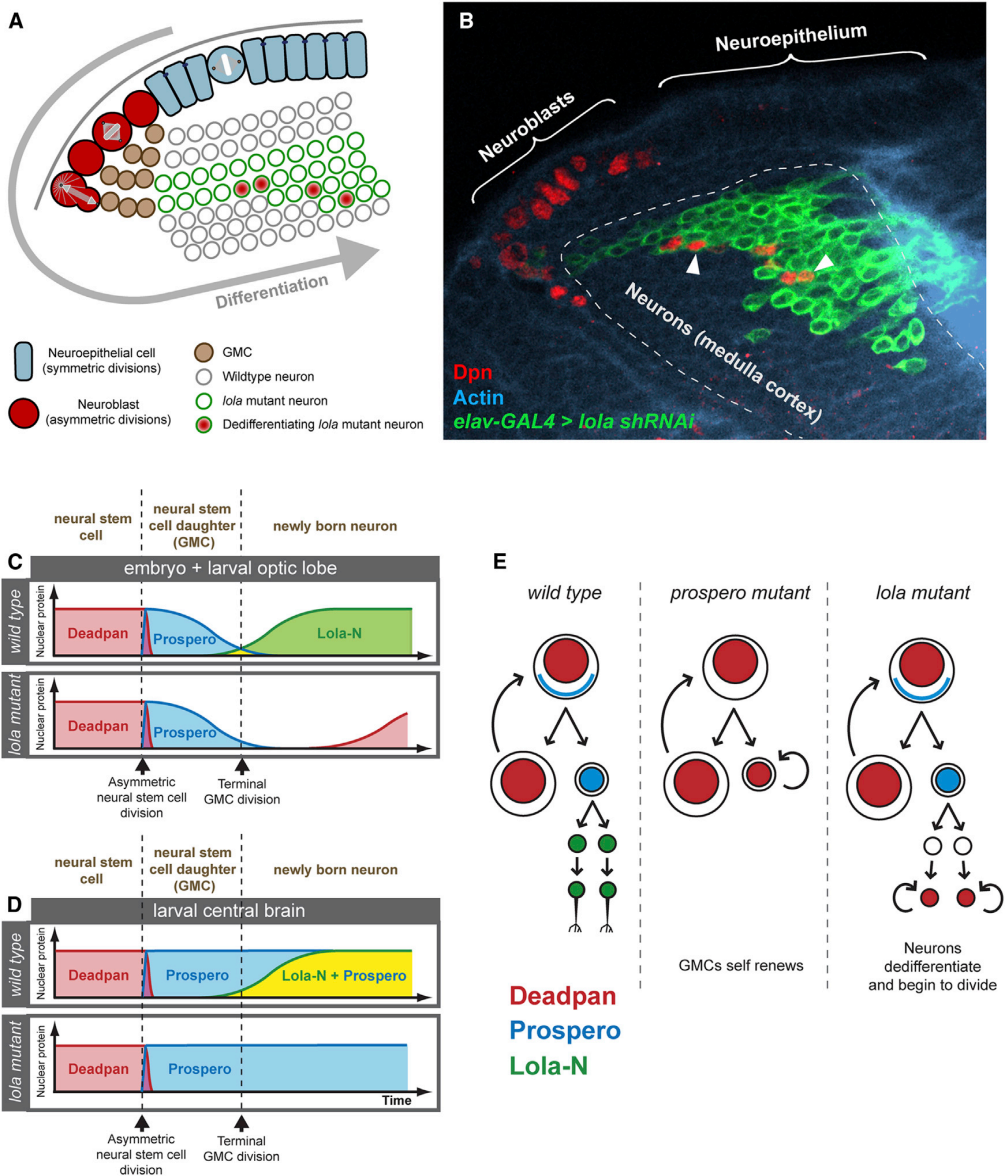
*lola* Mutant Neurons Dedifferentiate to a Neural Stem Cell Fate (A and B) Knockdown of *lola* in the larval optic lobe medulla cortex causes neurons to dedifferentiate and express Deadpan (arrowheads in B). MARCM clones expressing *lola shRNAi* in a subset of neurons are labeled in green. (C) Schematic diagram showing the temporal expression of Deadpan, Prospero, and Lola-N during neuronal differentiation in embryos and the developing optic lobe. (D) Temporal expression of Deadpan, Prospero, and Lola-N during neuronal differentiation in the larval central brain. (E) In the *wild-type* nervous system Prospero turns off neural stem cell genes, such as Deadpan, in GMCs. In *prospero* mutants, neural stem cell genes continue to be expressed in GMCs, leading to overproliferation and tumors. In *lola* mutants, Prospero represses neural stem cell genes in the GMCs. However, in neurons, without Lola to maintain repression, neural stem cell genes are reexpressed, and the cells dedifferentiate and form tumors. See also Figure S6.

Knockdown of *lola* in neurons (*elav-GAL4*-driven expression of a shRNA; Ni et al., 2011) results in dedifferentiation of neurons in the optic lobe and the formation of tumors in the adult brain (Figures 7B and Figure S6A). *elav-GAL4* was reported to be expressed weakly in neuroblasts (Berger et al., 2007). To exclude the possibility that knockdown of *lola* in neuroblasts contributes to the tumor phenotype, we drove *lola shRNAi* with *GAL4*^*C855a*^ (Egger et al., 2007). *GAL4*^*C855a*^ is expressed in the neuroepithelium, in optic lobe neuroblasts and in GMCs (Figures S6C–S6E). We never see tumors when *lola* is knocked down in these cell types. Therefore, tumors arise only when *lola* is knocked down in neurons. In addition, there is little or no expression of *elav-GAL4* in neuroblasts in our clonal experiments (see Figure 7B). We conclude that *lola* mutant neurons dedifferentiate, express stem cell genes, and proliferate giving rise to brain tumors in the adult.

## Discussion

Our results reveal the temporal progression toward neuronal differentiation. First, Prospero enters the nucleus of the newly born GMC and initiates the repression of neural stem cell genes. Next, Lola-N is expressed and maintains transcriptional repression in postmitotic neurons, acting as a differentiation “lock” (Figures 7C and 7D). Transcription factors that act as differentiation locks have been identified in other cell types (e.g., Pax5 in B cells; Cobaleda et al., 2007), but not in neurons. Mutations like *midlife crisis* (Carney et al., 2013), which lead to the transient derepression of neuroblast genes, are insufficient to cause neurons to dedifferentiate and revert to a proliferating stem-cell-like state, nor do they result in tumorigenesis.

We show that Lola-N is a potent repressor of neural stem cell genes. Studies on vertebrate BTB-ZFs have revealed that they act predominantly as transcriptional repressors (for review, see Kelly and Daniel, 2006), although some, such as Miz-1, can act as both repressors and activators (Adhikary et al., 2003). For several BTB-ZFs, transcriptional repression is elicited through HDACs, which deacetylate histones and promote a “closed” chromatin state (Kelly and Daniel, 2006). Therefore, as found for vertebrate BTB-ZFs, Lola-N may act through HDACs to achieve this repression.

The vertebrate proteins most similar to Lola are Zfp131, Miz-1, and Leukemia-Related Factor (LRF). Of these, Zfp131 is expressed predominantly in the developing nervous system, the adult brain, and the testes (Trappe et al., 2002), a similar expression pattern to that described for *lola-N* in flies (FlyAtlas; Chintapalli et al., 2007). Miz-1 is also expressed in neurons in the developing and adult mouse brain (Allen Brain Atlas; Lein et al., 2007) and has a potent growth arrest function (Peukert et al., 1997). Zfp131 and Miz-1 may be functionally analogous to Lola-N, with respect to promoting or maintaining neuronal differentiation.

That Lola-N represses cell-cycle genes in postmitotic neurons seems surprising at first; however, there is a growing body of evidence to suggest that neurons must continuously keep the cell cycle in check (for review, see Herrup and Yang, 2007). Inhibition of Retinoblastoma (Rb) in Purkinje neurons forces neurons to reenter the cell cycle and replicate their DNA, but M phase is not initiated and the neurons die (Feddersen et al., 1995). Similarly, knockdown of Cdh1, which is required to prevent the accumulation of cyclin B1 in neurons, causes neurons to enter S phase and leads to apoptosis (Almeida et al., 2005). Therefore, repression of cell-cycle genes is imperative as aberrant cell-cycle activity in neurons can lead to neurodegeneration or cancer.

Growing evidence suggests that there is plasticity between stem cells and their more differentiated progeny (Gupta et al., 2009) and that some more differentiated cells, for example, intestinal secretory progenitors (van Es et al., 2012) and intestinal epithelial cells (Schwitalla et al., 2013), can initiate tumors. In *lola* mutants, the tumor cells of origin are postmitotic neurons rather than GMCs (Figure 7E). In contrast to *prospero* mutants, GMCs differentiate normally in *lola* mutants, and Deadpan is repressed. Newly born neurons, however, dedifferentiate, reexpress Deadpan, and undergo cell division resulting in adult brain tumors.

Interestingly, the loss of *lola* does not cause tumor formation in the central brain, where Prospero expression persists in neurons. The diagrams in Figures 7C and 7D depict the expression of Deadpan, Prospero, and Lola-N, during neurogenesis in *wild-type* and *lola* mutants. In *lola* mutant embryos and larval optic lobe clones, Prospero is switched off in neurons and Deadpan is reexpressed (Figure 7C). However, in the larval central brain, Prospero remains on in neurons (Figure 7D). We have shown that Prospero expression can rescue the *lola* tumor phenotype (Figures 4E and 4F); therefore, the persistence of Prospero in central brain neurons may explain why Deadpan is not reexpressed and why tumors are not observed in the adult central brain.

We hypothesize that Lola maintains the repression of neural stem cell genes until they transition to a more permanent “off state.” BTB-zinc finger proteins are known to recruit HDACs, and this may be the mechanism by which target genes are more permanently locked down. In support of this hypothesis, we find that knockdown of *lola* in adult flies is no longer sufficient to cause neuronal dedifferentiation (Figure S6B).

A long-term goal of regenerative medicine and stem cell research is to convert cells in vivo to specific fates to allow for the repair of damaged or diseased tissues. The ability to induce neurons to dedifferentiate, followed by directed differentiation to neurons of choice, would be an ideal method of repair. Mature neurons have been shown to dedifferentiate when p53 and NF1 are knocked down simultaneously. However, this leads to genome instability resulting in gliomas (Friedmann-Morvinski et al., 2012). Our data suggest that a single factor can maintain the global repression of both cell-cycle genes and neural stem cell genes in postmitotic neurons. Factors such as Lola would be excellent targets for realizing the goal of controlled dedifferentiation of neurons in vivo.

## Experimental Procedures

### Fly Lines

*UAS-lola-N*, *UAS-lola-H*, *UAS-lola-F*, and *UAS-Dam-lola-N* flies were generated by PCR amplifying the full coding sequences from an embryonic cDNA library and cloning it into pUASTattB (Bischof et al., 2007) and pUAST-NDam (Choksi et al., 2006), respectively (for primer sequences, see Supplemental Experimental Procedures). Transgenic flies were generated as previously described (Choksi et al., 2006). *UAS-lola-shRNAi* (based on the approach described by Ni et al., 2011) was generated using the method available on the TRiP website (http://www.flyrnai.org/supplement/2ndGenProtocol.pdf) using the passenger strand sequence CACGACAGATCTCAGGATGAA and the pWALIUM20 vector. MARCM clones were generated using the following driver lines: *elav-GAL4*, *UAS-mCD8-GFP*, *hsFLP*; *FRT42D*, *tub-Gal80/CyO* and *tub-GAL4*, *UAS-nuGFP*, *hsFLP*; *FRT42D*, *tub-Gal80/CyO*, and the following FRT lines: *lola*^*E76*^, *FRT42D/CyO*, *lola*^*5D2*^, *FRT42D/CyO*, *lola*^*E76*^, *FRT42D/CyO*; *UAS-lola-N/TMBb*, and *FRT42D*; *UAS-lola-shRNAi*. *lola*^*E76*^ is a protein null mutation, removing all isoforms of *lola* (Goeke et al., 2003). *lola*^*5D2*^ (P-element insertion at the transcriptional start site) is a strong hypomorph for *lola* (Giniger et al., 1994). *w*^*1118*^ were used as *wild-type* flies for immunohistochemistry experiments.

### Yeast One-Hybrid

The yeast one-hybrid assay was performed using the Matchmaker Yeast One-Hybrid kit (Clontech) and protocol. To make the DNA bait construct, we first modified the Clontech bait vector pHis2.1 to make it compatible with our cDNA library by replacing the Trp selection gene with the Leu selection gene to give pHis2.2. An oligo with six copies of the bait site interspersed with random 5-mer (Supplemental Experimental Procedures) was annealed to its reverse complement and cloned into pHis2.2 using MluI and SpeI. This was transformed into Y187 yeast. One hundred micrograms of a 4–17 hr embryonic cDNA library (with cDNAs fused to an activator domain) was transformed into yeast carrying the bait construct and plated onto −His/−Leu/−Trp selective media containing 50 mM 3-AT. Positive colonies were picked, suspended in 0.2% SDS, heated at 95°C for 5 min, and centrifuged, and the supernatant was purified using a QIAGEN PCR purification kit. For sequencing, the cDNA inserts were PCR amplified (for primer sequences, see Supplemental Experimental Procedures) and sequenced using the forward primer.

### Immunohistochemistry

Larval and adult CNS were dissected in PBS and then fixed for 15–20 min in PBS containing 4% formaldehyde (ultra pure), 0.5 mM EGTA, and 5 mM MgCl_2_. Wash solution was PBS with 0.3% Triton X-100. The anti-Lola-N antibody was generated by synthesis of the peptides GVELDSIDDTMTEV and GSPLSWTYDAVKIC (corresponding to the unique C-terminal region) and injection into rabbits (Moravian Biotechnology). Serum was purified using the GVELDSIDDTMTEV peptide and used at a concentration of 1:10. Other primary antibodies used were chicken anti-GFP (1 in 2,000) (ab13970, Abcam), mouse anti-Discs Large (c) (1 in 70) (4F3, Developmental Studies Hybridoma Bank [DSHB]), rat anti-Elav (c) (1 in 70) (7E8A10, DSHB), mouse anti-Fas II (c) (1 in 20) (1D4, DSHB), guinea pig anti-Deadpan (1 in 500) (J.B. Skeath), mouse anti-Repo (c) (1 in 70) (8D12, DSHB), rabbit anti-PH3 (1 in 100) (06-570, Upstate), rat anti-PH3 (1 in 150) (ab10543, Abcam), mouse anti-Prospero (c) (1 in 70) (MR1A, DSHB), mouse anti-Cut (c) (1 in 30) (2B10, DSHB), rat anti-Worniu (0.8 in 1) (C.Q. Doe), rabbit anti-Asense (1 in 1000) (Y. N. Jan). Appropriate combinations of Alexa-coupled secondary antibodies (Invitrogen) were subsequently applied. Phalloidin-546 (Invitrogen) was used for actin staining (1 in 100). Samples were analyzed with a Leica SP2, Leica SP5, or Zeiss LSM510 confocal microscope. Adobe Photoshop and Illustrator were used to generate figures.

### DamID

Preparation of Dam-methylated DNA from stage 10–11 embryos was performed as previously described (Choksi et al., 2006). The Dam-only and Dam-Lola-N samples were labeled and hybridized together on a whole genome 2.1 million feature tiling array, with 50- to 75-mer oligonucleotides spaced at approximately 55 bp intervals (Nimblegen systems). Arrays were scanned and intensities extracted (Nimblegen Systems). Three biological replicates (with one dye-swap) were performed. Log2 ratios of each oligo were median normalized.

### DamID Analysis

A peak finding algorithm with FDR analysis was used to identify significant binding sites (Wolfram et al., 2012) (PERL script available on request). All peaks spanning four or more consecutive probes (greater than ∼900 bp for low-density arrays and greater than ∼400 bp for high-density arrays) over a 2-fold ratio change were assigned a FDR value.

### Motif Analysis

A perl program was used to identify peak structures within the DamID data (script available on request), and the top 1,000 peaks (based on peak height) were analyzed using the MICRA program (Southall and Brand, 2009). Lola-N data were first converted to release four coordinates before running MICRA. To generate a PWM from enriched 8-mer, the top 50 enriched 8-mer were analyzed by MEME-ChIP (Machanick and Bailey, 2011), with their abundance represented in the input fasta file.

### In Situ Hybridizations

In situ hybridization was performed as previously described (Choksi et al., 2006). For primer sequences used to generate in situ probes, see the Supplemental Experimental Procedures.

## Accession Numbers

The DamID-chip data have been deposited in the National Center for Biotechnology Information Gene Expression Omnibus under accession number GSE53447.

## Supplemental Information

Document S1. Supplemental Experimental Procedures and Figures S1–S6

## References

[bib1] Adhikary S., Peukert K., Karsunky H., Beuger V., Lutz W., Elsässer H.P., Möröy T., Eilers M. (2003). Miz1 is required for early embryonic development during gastrulation. Mol. Cell. Biol..

[bib2] Almeida A., Bolaños J.P., Moreno S. (2005). Cdh1/Hct1-APC is essential for the survival of postmitotic neurons. J. Neurosci..

[bib3] Bello B., Reichert H., Hirth F. (2006). The brain tumor gene negatively regulates neural progenitor cell proliferation in the larval central brain of Drosophila. Development.

[bib4] Berger C., Renner S., Lüer K., Technau G.M. (2007). The commonly used marker ELAV is transiently expressed in neuroblasts and glial cells in the Drosophila embryonic CNS. Dev. Dyn..

[bib5] Betschinger J., Mechtler K., Knoblich J.A. (2006). Asymmetric segregation of the tumor suppressor brat regulates self-renewal in Drosophila neural stem cells. Cell.

[bib6] Bischof J., Maeda R.K., Hediger M., Karch F., Basler K. (2007). An optimized transgenesis system for Drosophila using germ-line-specific phiC31 integrases. Proc. Natl. Acad. Sci. USA.

[bib7] Bowman S.K., Rolland V., Betschinger J., Kinsey K.A., Emery G., Knoblich J.A. (2008). The tumor suppressors Brat and Numb regulate transit-amplifying neuroblast lineages in Drosophila. Dev. Cell.

[bib8] Bryne J.C., Valen E., Tang M.H., Marstrand T., Winther O., da Piedade I., Krogh A., Lenhard B., Sandelin A. (2008). JASPAR, the open access database of transcription factor-binding profiles: new content and tools in the 2008 update. Nucleic Acids Res..

[bib9] Carney T.D., Struck A.J., Doe C.Q. (2013). midlife crisis encodes a conserved zinc-finger protein required to maintain neuronal differentiation in Drosophila. Development.

[bib10] Caussinus E., Gonzalez C. (2005). Induction of tumor growth by altered stem-cell asymmetric division in Drosophila melanogaster. Nat. Genet..

[bib11] Caussinus E., Hirth F. (2007). Asymmetric stem cell division in development and cancer. Prog. Mol. Subcell. Biol..

[bib12] Charest-Marcotte A., Dufour C.R., Wilson B.J., Tremblay A.M., Eichner L.J., Arlow D.H., Mootha V.K., Giguère V. (2010). The homeobox protein Prox1 is a negative modulator of ERRalpha/PGC-1alpha bioenergetic functions. Genes Dev..

[bib13] Chintapalli V.R., Wang J., Dow J.A. (2007). Using FlyAtlas to identify better Drosophila melanogaster models of human disease. Nat. Genet..

[bib14] Choksi S.P., Southall T.D., Bossing T., Edoff K., de Wit E., Fischer B.E., van Steensel B., Micklem G., Brand A.H. (2006). Prospero acts as a binary switch between self-renewal and differentiation in Drosophila neural stem cells. Dev. Cell.

[bib15] Cobaleda C., Jochum W., Busslinger M. (2007). Conversion of mature B cells into T cells by dedifferentiation to uncommitted progenitors. Nature.

[bib16] Cook T., Pichaud F., Sonneville R., Papatsenko D., Desplan C. (2003). Distinction between color photoreceptor cell fates is controlled by Prospero in Drosophila. Dev. Cell.

[bib17] Doe C.Q. (2008). Neural stem cells: balancing self-renewal with differentiation. Development.

[bib18] Doe C.Q., Chu-LaGraff Q., Wright D.M., Scott M.P. (1991). The prospero gene specifies cell fates in the Drosophila central nervous system. Cell.

[bib19] Egger B., Boone J.Q., Stevens N.R., Brand A.H., Doe C.Q. (2007). Regulation of spindle orientation and neural stem cell fate in the Drosophila optic lobe. Neural Dev..

[bib20] Egger B., Chell J.M., Brand A.H. (2008). Insights into neural stem cell biology from flies. Philos. Trans. R. Soc. Lond. B Biol. Sci..

[bib21] Feddersen R.M., Clark H.B., Yunis W.S., Orr H.T. (1995). In vivo viability of postmitotic Purkinje neurons requires pRb family member function. Mol. Cell. Neurosci..

[bib22] Friedmann-Morvinski D., Bushong E.A., Ke E., Soda Y., Marumoto T., Singer O., Ellisman M.H., Verma I.M. (2012). Dedifferentiation of neurons and astrocytes by oncogenes can induce gliomas in mice. Science.

[bib23] Giniger E., Tietje K., Jan L.Y., Jan Y.N. (1994). lola encodes a putative transcription factor required for axon growth and guidance in Drosophila. Development.

[bib24] Goeke S., Greene E.A., Grant P.K., Gates M.A., Crowner D., Aigaki T., Giniger E. (2003). Alternative splicing of lola generates 19 transcription factors controlling axon guidance in Drosophila. Nat. Neurosci..

[bib25] Gupta P.B., Chaffer C.L., Weinberg R.A. (2009). Cancer stem cells: mirage or reality?. Nat. Med..

[bib26] Gurdon J.B. (1962). The developmental capacity of nuclei taken from intestinal epithelium cells of feeding tadpoles. J. Embryol. Exp. Morphol..

[bib27] Herrup K., Yang Y. (2007). Cell cycle regulation in the postmitotic neuron: oxymoron or new biology?. Nat. Rev. Neurosci..

[bib28] Kelly K.F., Daniel J.M. (2006). POZ for effect—POZ-ZF transcription factors in cancer and development. Trends Cell Biol..

[bib29] Kristiansen L.V., Hortsch M. (2010). Fasciclin II: the NCAM ortholog in Drosophila melanogaster. Adv. Exp. Med. Biol..

[bib30] Lee T., Luo L. (2001). Mosaic analysis with a repressible cell marker (MARCM) for Drosophila neural development. Trends Neurosci..

[bib31] Lee C.Y., Andersen R.O., Cabernard C., Manning L., Tran K.D., Lanskey M.J., Bashirullah A., Doe C.Q. (2006). Drosophila Aurora-A kinase inhibits neuroblast self-renewal by regulating aPKC/Numb cortical polarity and spindle orientation. Genes Dev..

[bib32] Lee C.Y., Wilkinson B.D., Siegrist S.E., Wharton R.P., Doe C.Q. (2006). Brat is a Miranda cargo protein that promotes neuronal differentiation and inhibits neuroblast self-renewal. Dev. Cell.

[bib33] Lee S., Kang J., Yoo J., Ganesan S.K., Cook S.C., Aguilar B., Ramu S., Lee J., Hong Y.K. (2009). Prox1 physically and functionally interacts with COUP-TFII to specify lymphatic endothelial cell fate. Blood.

[bib34] Lein E.S., Hawrylycz M.J., Ao N., Ayres M., Bensinger A., Bernard A., Boe A.F., Boguski M.S., Brockway K.S., Byrnes E.J. (2007). Genome-wide atlas of gene expression in the adult mouse brain. Nature.

[bib35] Liu Y.W., Gao W., Teh H.L., Tan J.H., Chan W.K. (2003). Prox1 is a novel coregulator of Ff1b and is involved in the embryonic development of the zebra fish interrenal primordium. Mol. Cell. Biol..

[bib36] Machanick P., Bailey T.L. (2011). MEME-ChIP: motif analysis of large DNA datasets. Bioinformatics.

[bib37] Matsuzaki F., Koizumi K., Hama C., Yoshioka T., Nabeshima Y. (1992). Cloning of the Drosophila prospero gene and its expression in ganglion mother cells. Biochem. Biophys. Res. Commun..

[bib38] Neumüller R.A., Knoblich J.A. (2009). Dividing cellular asymmetry: asymmetric cell division and its implications for stem cells and cancer. Genes Dev..

[bib39] Neumüller R.A., Richter C., Fischer A., Novatchkova M., Neumüller K.G., Knoblich J.A. (2011). Genome-wide analysis of self-renewal in Drosophila neural stem cells by transgenic RNAi. Cell Stem Cell.

[bib40] Ni J.Q., Zhou R., Czech B., Liu L.P., Holderbaum L., Yang-Zhou D., Shim H.S., Tao R., Handler D., Karpowicz P. (2011). A genome-scale shRNA resource for transgenic RNAi in Drosophila. Nat. Methods.

[bib41] Ohsako T., Horiuchi T., Matsuo T., Komaya S., Aigaki T. (2003). Drosophila lola encodes a family of BTB-transcription regulators with highly variable C-terminal domains containing zinc finger motifs. Gene.

[bib42] Pasque V., Jullien J., Miyamoto K., Halley-Stott R.P., Gurdon J.B. (2011). Epigenetic factors influencing resistance to nuclear reprogramming. Trends Genet..

[bib43] Peukert K., Staller P., Schneider A., Carmichael G., Hänel F., Eilers M. (1997). An alternative pathway for gene regulation by Myc. EMBO J..

[bib44] Rhyu M.S., Jan L.Y., Jan Y.N. (1994). Asymmetric distribution of numb protein during division of the sensory organ precursor cell confers distinct fates to daughter cells. Cell.

[bib45] Schwitalla S., Fingerle A.A., Cammareri P., Nebelsiek T., Göktuna S.I., Ziegler P.K., Canli O., Heijmans J., Huels D.J., Moreaux G. (2013). Intestinal tumorigenesis initiated by dedifferentiation and acquisition of stem-cell-like properties. Cell.

[bib46] Seeger M., Tear G., Ferres-Marco D., Goodman C.S. (1993). Mutations affecting growth cone guidance in Drosophila: genes necessary for guidance toward or away from the midline. Neuron.

[bib47] Shan S.F., Wang L.F., Zhai J.W., Qin Y., Ouyang H.F., Kong Y.Y., Liu J., Wang Y., Xie Y.H. (2008). Modulation of transcriptional corepressor activity of prospero-related homeobox protein (Prox1) by SUMO modification. FEBS Lett..

[bib48] Southall T.D., Brand A.H. (2009). Neural stem cell transcriptional networks highlight genes essential for nervous system development. EMBO J..

[bib49] Spana E.P., Doe C.Q. (1995). The prospero transcription factor is asymmetrically localized to the cell cortex during neuroblast mitosis in Drosophila. Development.

[bib50] Steffensen K.R., Holter E., Båvner A., Nilsson M., Pelto-Huikko M., Tomarev S., Treuter E. (2004). Functional conservation of interactions between a homeodomain cofactor and a mammalian FTZ-F1 homologue. EMBO Rep..

[bib51] Takahashi K., Yamanaka S. (2006). Induction of pluripotent stem cells from mouse embryonic and adult fibroblast cultures by defined factors. Cell.

[bib52] Tea J.S., Chihara T., Luo L. (2010). Histone deacetylase Rpd3 regulates olfactory projection neuron dendrite targeting via the transcription factor Prospero. J. Neurosci..

[bib53] Trappe R., Buddenberg P., Uedelhoven J., Gläser B., Buck A., Engel W., Burfeind P. (2002). The murine BTB/POZ zinc finger gene Znf131: predominant expression in the developing central nervous system, in adult brain, testis, and thymus. Biochem. Biophys. Res. Commun..

[bib54] Vaessin H., Grell E., Wolff E., Bier E., Jan L.Y., Jan Y.N. (1991). prospero is expressed in neuronal precursors and encodes a nuclear protein that is involved in the control of axonal outgrowth in Drosophila. Cell.

[bib55] van Es J.H., Sato T., van de Wetering M., Lyubimova A., Nee A.N., Gregorieff A., Sasaki N., Zeinstra L., van den Born M., Korving J. (2012). Dll1+ secretory progenitor cells revert to stem cells upon crypt damage. Nat. Cell Biol..

[bib56] Vierbuchen T., Ostermeier A., Pang Z.P., Kokubu Y., Südhof T.C., Wernig M. (2010). Direct conversion of fibroblasts to functional neurons by defined factors. Nature.

[bib57] Waddington C.H. (1957). The Strategy of Genes: A Discussion of Some Aspects of Theoretical Biology.

[bib58] Wang H., Somers G.W., Bashirullah A., Heberlein U., Yu F., Chia W. (2006). Aurora-A acts as a tumor suppressor and regulates self-renewal of Drosophila neuroblasts. Genes Dev..

[bib59] Wodarz A., Gonzalez C. (2006). Connecting cancer to the asymmetric division of stem cells. Cell.

[bib60] Wolfram V., Southall T.D., Brand A.H., Baines R.A. (2012). The LIM-homeodomain protein islet dictates motor neuron electrical properties by regulating K(+) channel expression. Neuron.

[bib61] Yousef M.S., Matthews B.W. (2005). Structural basis of Prospero-DNA interaction: implications for transcription regulation in developing cells. Structure.

[bib62] Zheng L., Carthew R.W. (2008). Lola regulates cell fate by antagonizing Notch induction in the Drosophila eye. Mech. Dev..

