# Supplementary material for: Dedifferentiation of Neurons Precedes Tumor Formation in lola Mutants
Source: Dev Cell. 2014 Mar 31;28(6):685–96. doi: 10.1016/j.devcel.2014.01.030 (PMC3978655; doi:10.1016/j.devcel.2014.01.030)
Supplement: Document S1. Supplemental Experimental Procedures and Figures S1–S6 [file mmc1.pdf]

Developmental Cell, Volume *28*

Supplemental Information

Dedifferentiation of Neurons Precedes Tumor Formation in *lola* Mutants

Tony D. Southall, Catherine M. Davidson, Claire Miller, Adrian Carr, and Andrea H. Brand

Supplemental Figures

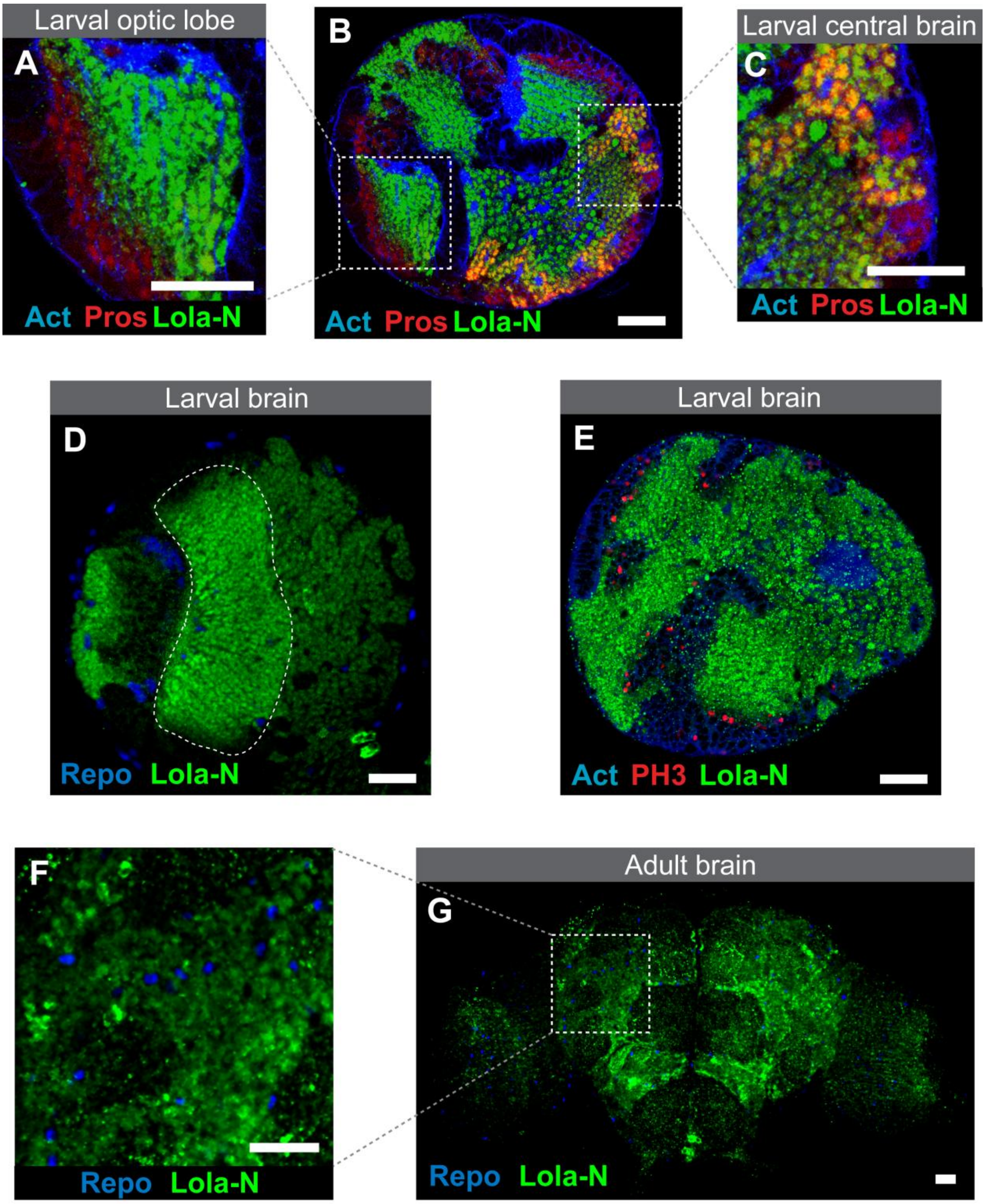

Figure S1, related to Figure 2. Lola-N expression in the larval and adult brain. (A-C) Magnified views of Lola-N and Prospero expression patterns in the larval optic lobe and central brain (from Figure 2G). (A) In the optic lobe, there is very little overlap between Prospero and Lola-N expression, similar to the embryo (Figure 2E). (C) In the central brain, many neurons express both Prospero and Lola-N. (D) Lola-N is expressed in medulla cortex neurons (outlined). (E) Similar to the embryo (Figure 2F), Lola-N is expressed in post-mitotic cells and does not colocalise with PH3. (F-G) Lola-N expression in the adult brain. Although the Lola-N antibody does not perform optimally for adult brain staining, Lola-N is clearly present in the majority, if not all, neurons and is absent from glia (the adult brain consists of only two cell types, neurons and glia). Scale bars represent 20 μM.

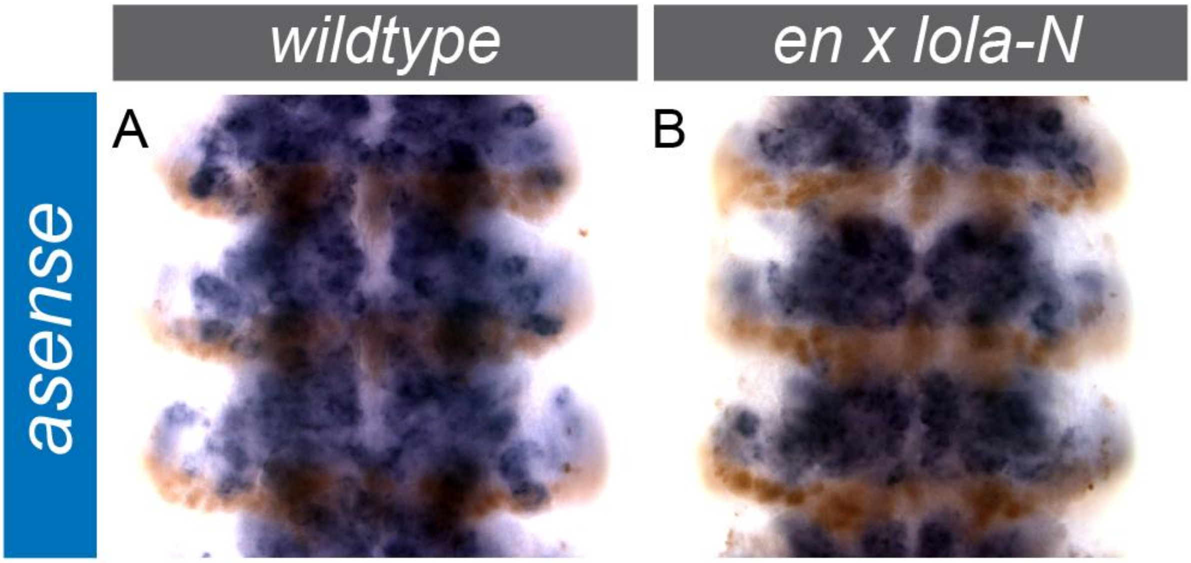

Figure S2, related to Figure 3. Lola-N represses *asense* transcription in the embryo. (A) *In situ* hybridization of *asense* mRNA (blue) in wildtype stage 10-11 embryos and (B) in embryos expressing Lola-N in engrailed stripes. *engrailed* (*en*) expression pattern is shown in brown.

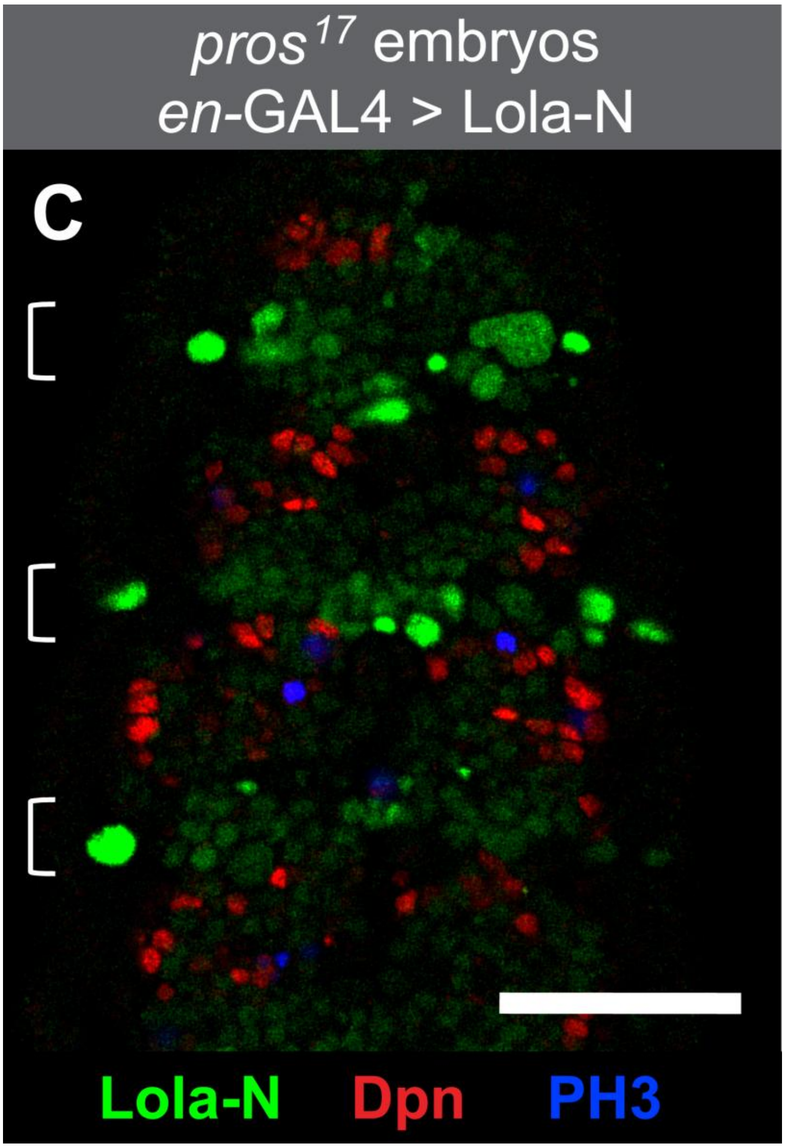

Figure S3, related to Figure 4. Lola-N rescues the *prospero* phenotype. Lola-N represses neuroblast genes and proliferation in *prospero* mutants. Driving expression of Lola-N in *prospero* mutant embryos with *engailed*-GAL4 (stripes, see white brackets) represses expression of Deadpan and prevents cell division (as assayed by PH3). No Deadpan or PH3 is observed in any cells expressing Lola-N. Ventral view of a stage 17 embryo. Scale bar represents 20 µM.

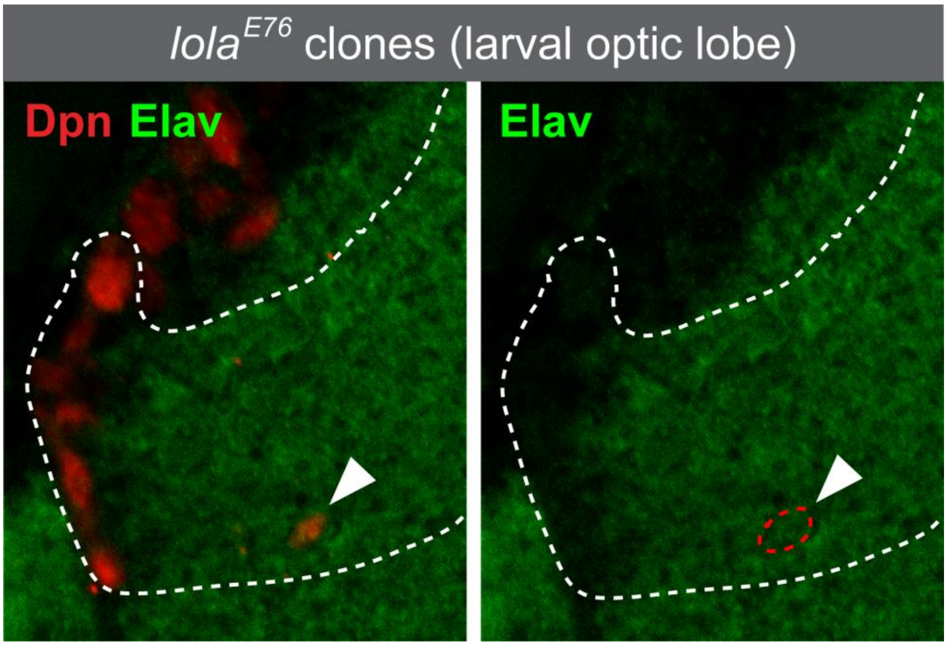

Figure S4, related to Figure 5. Elav and Deadpan colocalise in dedifferentiating *lola*^{E76} neurons of the larval optic lobe medulla cortex (see arrowhead). The clone is outlined by the dashed line. Note that neuroblasts do not show expression of Elav.

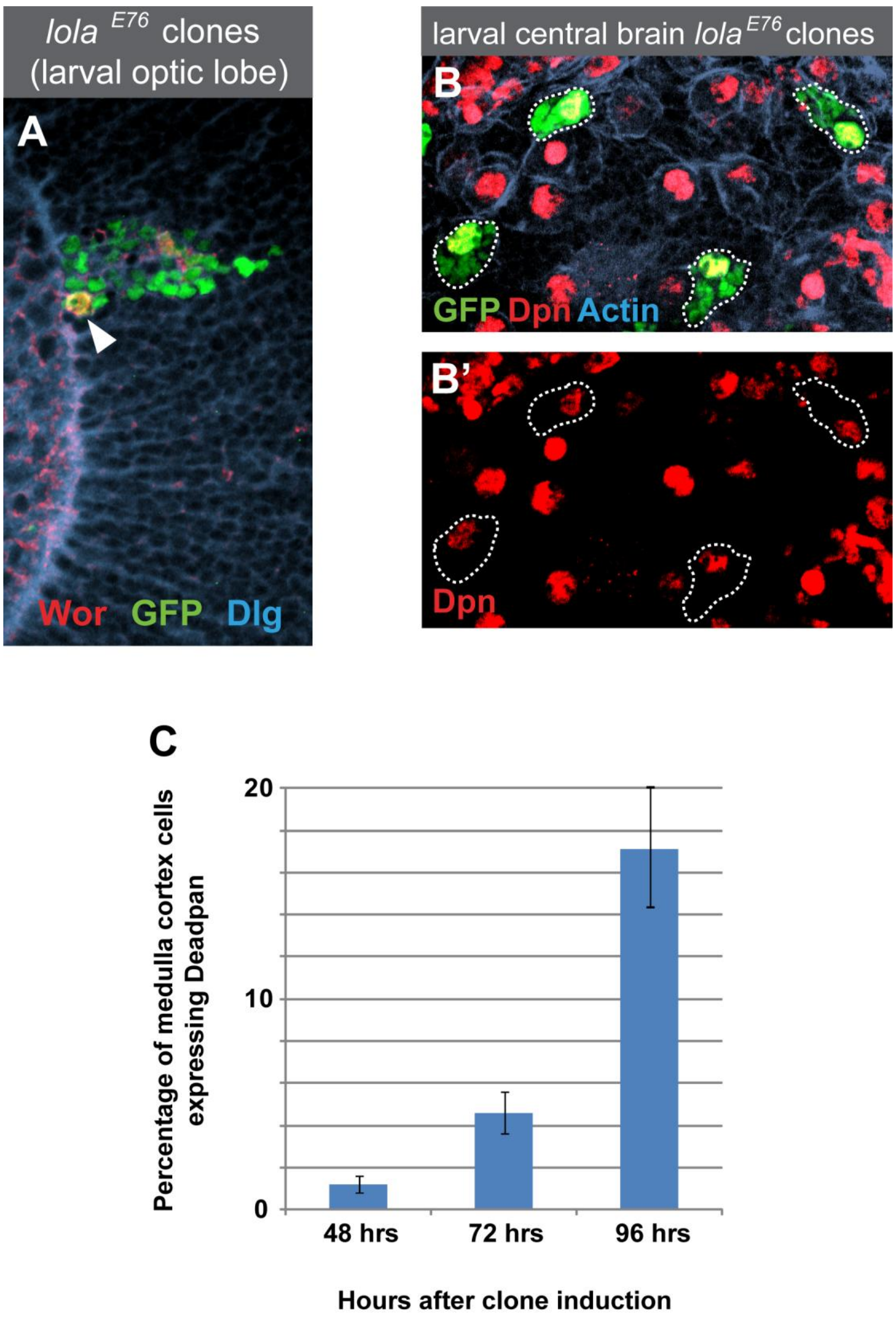

Figure S5, related to Figure 6. (A) Dedifferentiating *lola*^{E76} neurons in the optic lobe medulla cortex express Worniu (see arrowhead). (B) Larval central brain *lola*^{E76} clones do not exhibit ectopic Deadpan ((B') just the Deadpan channel). Single *lola*^{E76} clones contain one large Deadpan positive cell (neuroblast) and all progeny are Deadpan negative. (C) Quantification of the number of neurons ectopically expressing Deadpan in the medulla cortex after *lola*^{E76} clone induction. Error bars show standard deviation.

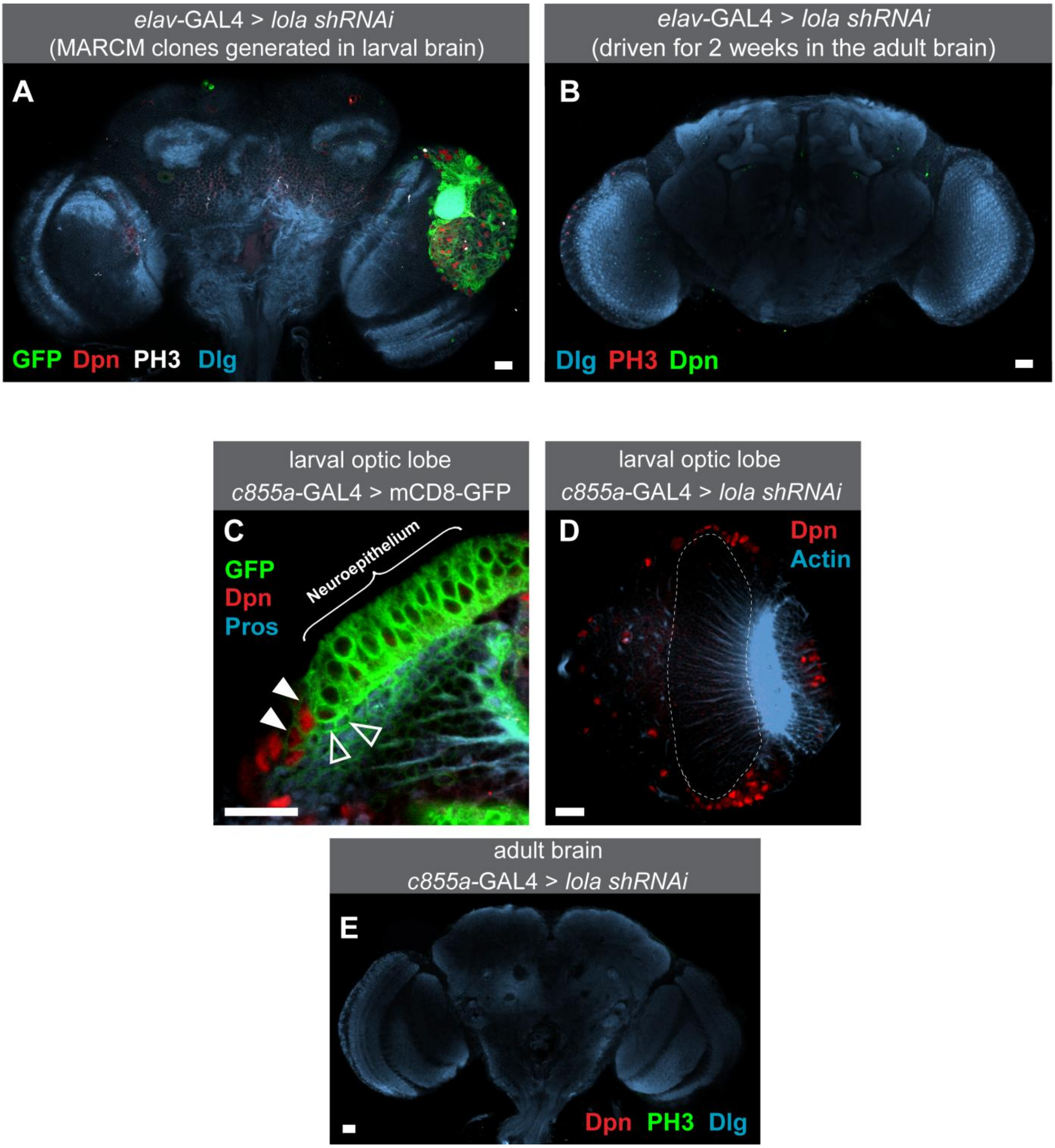

Figure S6, related to Figure 7. (A) Knock-down of *lola* in neurons results in tumours in the adult brain. (A) MARCM clones were generated during the 2nd instar larval stage that result in the expression of *lola shRNAi* in neurons (*elav*-GAL4). (B) Expression of *lola shRNAi* in all neurons of the adult brain from 2 days after eclosion, for 2 weeks, does not result in tumours. (C,D and E) Knock-down of *lola* in the neuroepithelium, neuroblasts and GMCs does not result in dedifferentiation or tumour formation. (C) *c855a*-GAL4 expression is observed in the neuroepithelium, newly born neuroblasts (closed arrowheads) and GMCs (as marked by nuclear Prospero, open arrowheads). (D) Ectopic expression of Deadpan is not observed in the optic lobe medulla cortex (outlined) when *lola* is knocked down (*lola shRNAi*) with *c855a*-GAL4. (E) No tumours are observed in the adult brain when *lola* is knocked down with *c855a*-GAL4. Scale bars represent 20 µM.

Supplemental Experimental Procedures

Primer name/description	Sequence (5' to 3')
LEU2 cassette forward	ATCCATGGACTCCAAGCTGCCTTTGTGTGC (with NcoI site)
LEU2 cassette reverse	GTACGCGGCCGCAGTATTGTTTGTGCACTTGCCCTAG (with NotI site)
Yeast one-hybrid bait sequence complement	GGACGCGTTTGTATAGAAAAGTTGATCGATCGACAGAATC GATCGTCGAAATCGATCGACGCGATCGATCGGCTCTATCG ATCGAGTGGATCGATCGCAAGTACTAGTCC
Prey cDNA amplify forward	AAACAAATTCTCAAGCGCTTTCAC
Prey cDNA amplify reverse	TTGACCCTTTTCCATCTTTTCGTA
lola-N CDS forward (with NotI site)	GTACGCGGCCGCTCATGGATGACGATCAGCAGTTTT GTTTG
lola-N CDS reverse (with XbaI site)	GTCTAGACTAAGCGCTATCGTTCTGGAACAC
lola-F CDS forward (with NotI site)	ATATGCGGCCGCATGGATGACGATCAGCAG
lola-F CDS reverse (with KpnI site)	CGGGTACCTTAGTTGTTGTTATAAGCAAATG
lola-H CDS forward (with EcoRI site)	CGAATTCATGGATGACGATCAGCAGTTTTG
lola-H CDS reverse (with NotI site)	ATATGCGGCCGCCTATGCGGTGGCCTCCCATTTG
string in situ probe forward	CTAAAATGCAATACTAGCCAAAAA
string in situ probe reverse	CAGTAATACGACTCACTATTACAATACGATAACACCCAAAC TTAG
CyclinE in situ probe forward	GTTGATCGAGGTCTGTGAAGTTTA
CyclinE in situ probe reverse	CAGTAATACGACTCACTATTAGTGACAATATTGGGGC AGATTAGT
asense in situ probe forward	GCTAAAAGTACACCCGCAAT
asense in situ probe reverse	CAGTAATACGACTCACTATTAAGGTAGCAGTGAGGCA TTTC
deadpan in situ probe forward	AGGTGAACCGCTATGTAAGC
deadpan in situ probe reverse	CAGTAATACGACTCACTATTAACTCCATCGAATTTTGC ATT

Primer sequences for cloning and in situ probes
